# No metagenomic evidence of tumorigenic viruses in cancers from a selected cohort of immunosuppressed subjects

**DOI:** 10.1038/s41598-019-56240-1

**Published:** 2019-12-24

**Authors:** Nunzia Passaro, Andrea Casagrande, Matteo Chiara, Bruno Fosso, Caterina Manzari, Anna Maria D’Erchia, Samuele Iesari, Francesco Pisani, Antonio Famulari, Patrizia Tulissi, Stefania Mastrosimone, Maria Cristina Maresca, Giuseppe Mercante, Giuseppe Spriano, Giacomo Corrado, Enrico Vizza, Anna Rosa Garbuglia, Maria Rosaria Capobianchi, Carla Mottini, Alessandra Cenci, Marco Tartaglia, Alessandro Nanni Costa, Graziano Pesole, Marco Crescenzi

**Affiliations:** 1 Italian National Institute of Health, Dept. of Cell Biology and Neurosciences, Rome, 00161 Italy; 2 Italian National Institute of Health, Dept. of Oncology and Molecular Medicine, Rome, 00161 Italy; 3 University of Milan, Dept. of Biosciences, Milan, 20122 Italy; 4 National Research Council, Institute of Biomembranes, Bioenergetics and Molecular Biotechnologies (IBIOM), Bari, 70126 Italy; 5 University of Bari “Aldo Moro”, Dept of Biosciences, Biotechnologies and Biopharmaceutics, Bari, 70124 Italy; 6 University of L’Aquila, Department of Biotechnological and Applied Clinical Sciences, L’Aquila, 67100 Italy; 7 Université catholique de Louvain, Pôle de Chirurgie Expérimentale et Transplantation, Institut de Recherche Expérimentale et Clinique, Brussels, 1200 Belgium; 8 ASUIUD S. Maria della Misericordia, Nephrology Unit, Udine, 33100 Italy; 9 ULSS 2, Nephrology, Dialysis, Transplantation Unit, Treviso, 31100 Italy; 10 Humanitas University, Department of Biomedical Sciences, Milan, 20090 Italy; 11 Humanitas Clinical and Research Center, Pieve Emanuele, MI 20090 Italy; 12 Regina Elena National Cancer Institute, Dept. of Experimental Clinical Oncology, Gynecologic Oncology Unit, Rome, 00144 Italy; 13 Fondazione Policlinico Universitario A. Gemelli IRCCS, Dept. of Women and Children Health, Gynecologic Oncology Unit, Rome, 00168 Italy; 14 National Institute for Infectious Diseases “L. Spallanzani” IRCCS, Laboratory of Virology, Rome, 00149 Italy; 15 Ospedale Pediatrico Bambino Gesù IRCCS, Genetics and Rare Diseases Research Division, Rome, 00165 Italy; 16 Italian National Institute of Health, National Transplant Center, Rome, 00162 Italy; 17 Italian National Institute of Health, Core Facilities, Rome, 00161 Italy

**Keywords:** Cancer genomics, Tumour virus infections

## Abstract

The possible existence of yet undiscovered human tumorigenic viruses is still under scrutiny. The development of large-scale sequencing technologies, coupled with bioinformatics techniques for the characterization of metagenomic sequences, have provided an invaluable tool for the detection of unknown, infectious, tumorigenic agents, as demonstrated by several recent studies. However, discoveries of novel viruses possibly associated with tumorigenesis are scarce at best. Here, we apply a rigorous bioinformatics workflow to investigate in depth tumor metagenomes from a small but carefully selected cohort of immunosuppressed patients. While a variegated bacterial microbiome was associated with each tumor, no evidence of the presence of putative oncoviruses was found. These results are consistent with the major findings of several recent papers and suggest that new human tumorigenic viruses are not common even in immunosuppressed populations.

## Introduction

The possible existence of human neoplasms of yet undiscovered infectious origin has long been the object of debate and persistent investigations^1^.

Detecting infectious agents responsible for human cancers is not a straightforward process. Epidemiological approaches are hindered by very long latencies between infections and the ensuing neoplasms and by low infection/tumor ratios^2^. Traditional microbiological approaches also meet formidable obstacles. Koch’s postulates cannot be applied since, in most cases, the causative agent does not reproduce in the cancer cells. Furthermore, in many instances the infectious agent contributes to tumorigenesis only indirectly, e.g., by promoting chronic inflammation and cell proliferation, and is not tightly associated with the tumor^2,3^.

In the last decade, Next Generation Sequencing (NGS) technologies have been widely used to study the dynamics that shape the evolution of cancer genomes. More recently, NGS has been applied also to the study of cancer metagenomics, with the aim of detecting novel, uncharacterized pathogenic agents, possibly associated with tumorigenesis. Bioinformatics strategies for the characterization of cancer metagenomes usually perform digital subtraction of human reads^4^ by alignment with the reference assembly of the human genome sequence. Then apply sophisticated approaches based on meta-genome assembly, quantification, and functional characterization of the presumably exogenous reads, in order to identify novel organisms associated with the tumor. This type of approach has been instrumental to the discovery of the Merkel cell polyomavirus, a previously unknown human virus believed to be responsible for most cutaneous Merkel cell carcinomas^5^. However, conceptually similar analyses of several thousands of tumors in the Cancer Genome Atlas^6^ have indicated that viruses are not likely to be involved in the tumorigenesis of 19 common cancers. Additional studies performed on populations with high cancer risk, though detecting a number of viruses possibly associated with cancer, have not yet pointed to any causative microorganism^7,8^. These largely negative findings cannot exclude that less common neoplasms and/or specific tumor subsets might still harbor known or unknown tumorigenic, infectious agents.

In an effort to identify potentially novel tumor-inducing microorganisms, we focused on immunosuppressed subjects. Organ transplant recipients, who routinely undergo immune suppression, and other naturally immunosuppressed patients, such as those affected by hematopoietic neoplasms, show a significantly increased incidence of specific tumor types, compared with the immunocompetent population^9^. In transplant acceptors, cancer incidence begins to rise several years after the beginning of immunosuppressive therapy^10^, suggestive of long incubation periods between possible infections and the insurgence of a clinically detectable neoplasm. Thus, it has been hypothesized that other tumors, also frequently occurring in immunosuppressed people, might be co-induced by infectious agents^2,10^. The competing/complementary immune surveillance theory posits that, in immunodeficient patients, tumors develop more frequently because the immune system is unable to suppress them^11^.

Here, we describe a metagenomics-based search for putative novel oncogenic microorganisms in tumors from a carefully selected cohort of immunodeficient subjects. We believe that the experimental strategy adopted in this study overcomes some of the limitations of previous investigations. Although two new strains of torque teno virus and a novel strain of coxsackievirus were identified in the 13 samples included in this study, we did not detect any viruses with plausible tumorigenic potential. Together with other reports in the literature, our findings suggest that, in the cancers we studied, directly-acting, oncogenic viruses are not common.

## Results

Tumor samples were obtained from patients that underwent therapeutic surgery. Only neoplasms diagnosed after at least three years from the onset of the immunosuppressive condition were considered, since it has been shown that tumor incidence begins to increase several years after kidney transplantation^10^. All tumor types selected for this study show higher incidences in immunosuppressed patients. All samples were collected in a sterile fashion in the operating room. To prevent exogenous contaminations, sterility was maintained up to and including extraction of nucleic acids. Table 1 summarizes the neoplasms analyzed and their provenance.

**Table 1:** Tumors analyzed.

Code	Tumor type	Nucleic acid sequenced	Immunosuppressive condition (IC)	Years from onset of IC
T1	Skin squamous cell carcinoma	RNA	Renal transplantation, immunosuppressive therapy	20
T2	Skin basal cell carcinoma	RNA	Renal transplantation, immunosuppressive therapy	9
T5	Native kidney (oncocytoma)	RNA	Renal transplantation, immunosuppressive therapy	19
T7	Transplanted kidney (clear cell carcinoma)	DNA and RNA	Renal transplantation, immunosuppressive therapy	3
T8	Native kidney (oncocytoma)	DNA	Renal transplantation, immunosuppressive therapy	20
T9	Non-Hodgkin Lymphoma	DNA	Renal transplantation, immunosuppressive therapy	12
T10	Colon adenocarcinoma	DNA	Renal transplantation, immunosuppressive therapy	5
T11	Native kidney (clear cell carcinoma)	RNA	Renal transplantation, immunosuppressive therapy	7
T12, T13	Two skin carcinomas	RNA	Renal transplantation, immunosuppressive therapy	12
T14	Skin squamous cell carcinoma	RNA	Renal transplantation, immunosuppressive therapy	8
N4	Carcinoma of the tongue and oropharynx	RNA	Non-Hodgkin lymphoma	15
N6	Lip squamous cell carcinoma (HPV-neg.)	RNA	Acute lymphocytic leukemia	11

**Figure 1:**
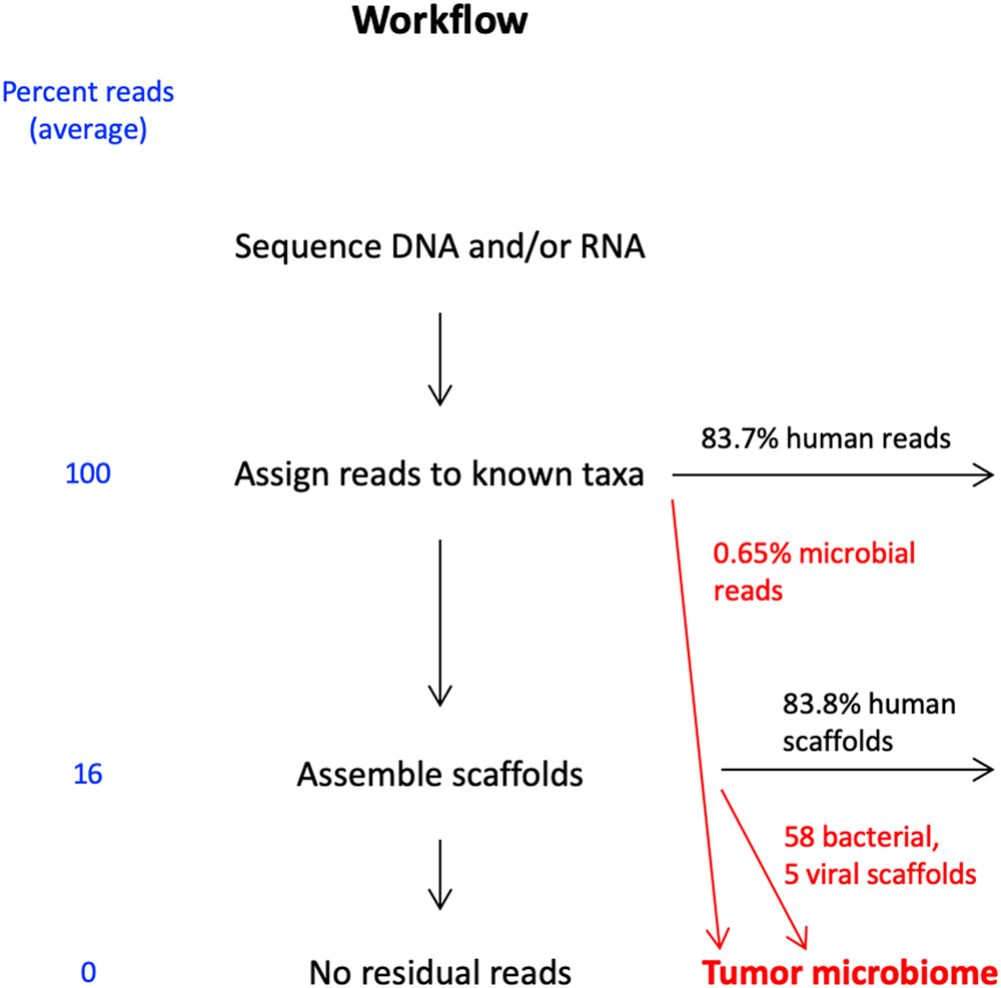
Schematic of the sequence analysis workflow.

Tumor RNA-Seq produced, on average, 96 million paired-end (PE) reads (range: 81–118 million) and DNA-Seq 98 million (range: 59–136 million), as reported in Table 2. DNA-Seq attained 3.8x–8.8x coverage of the human genome. RNA-Seq averaged a theoretical 10x coverage of RNAs (median length 2787 nt^12^) expressed at 1 copy/cell or 1x coverage of RNAs expressed at 1 copy/cell in tumor cells diluted in a 10-fold excess of non-tumor transcripts, assuming 500,000 transcripts/cell^13^. Taxonomic assignment of the reads, outlined in Fig. 1, was obtained by alignment to the reference human genome assembly and to a non-redundant collection of all the publicly available microbial genomic sequences integrated in the MetaShot tool (see below).

**Table 2:** Metagenomics analyses.

Code	Nucleic acid sequenced	Denoised PE reads analyzed^a^	Human-like scaffolds^b^	Notable findings
T1	RNA	109,426,939	98	Coxsackievirus (1 scaffold)
T2	RNA	100,344,056	43	
T5	RNA	80,678,185	43	
T7 _DNA_	DNA	101,083,848	664	
T7 _RNA_	RNA	90,253,628	1058	
T8	DNA	136,198,410	254	TTV (3 scaffolds)
T9	DNA	95,189,284	518	
T10	DNA	58,588,892	1246	
T11	RNA	105,289,212	1046	
T12	RNA	117,697,380	89	
T13	RNA	96,539,124	265	
N3	RNA	85,965,850	67	
N4	RNA	80,826,768	32	*F. nucleatum* (58 scaffolds)
N6	RNA	95,336,818	69	TTV (1 scaffold)

^a^Actual number of reads analyzed, after removing low-quality ones. ^b^Scaffolds are constructed by linking together a non-contiguous series of genomic sequences, consisting of sequences separated by gaps of known length; linked sequences are typically contiguous, corresponding to read overlaps.

In the early phases of this work, we found that existing bioinformatics tools performed suboptimally in some complex metagenomics tasks. For example, none of the tools considered was capable of correctly identifying human papillomavirus (HPV) in a uterine cervix carcinoma^14^. For these reasons, we devised a new workflow, MetaShot, that showed better performance in the classification of viral sequences^14^. The new tool correctly identified HPV31 in the uterine cervix carcinoma mentioned above^14^, while detecting no viruses in a confirmed HPV-negative clear cell carcinoma of the cervix (unpublished). Though partially reported elsewhere, the above results were obtained in the course of the present study, following exactly the same methods described here. Thus, they are witnesses to the sensitivity and specificity of our methods.

As analyzed with MetaShot, on average, 83.7% of the reads aligned unequivocally to the human genome and approximately 0.65% to microbial taxa (details on the latter in Supplementary Table S1). These percentages are relatively low because of the stringent assignment criteria adopted by MetaShot to avoid spurious calls.

Unassigned reads, i.e., reads that were not associated with any known taxon according to MetaShot, were assembled using the metaSPAdes^15^ metagenomic assembler. Subsequently, a simple strategy based on sequence similarity searches in publicly available databases (see Materials and Methods) was used to associate the resulting metagenomics scaffolds with closely related species or taxonomic groups. To exclude spurious assignments and consider only relatively abundant microorganisms in the assemblies, we arbitrarily decided to consider “detected” only organisms for which at least 1% of the total genome size or more than 10 kb of the genomic sequence was covered by metagenomics scaffolds. The vast majority (99.62%) of the scaffolds thus obtained were putatively assigned to a species. As outlined in Table 2, notable differences in the number of metagenomics scaffolds obtained from the different samples were observed. Strikingly, when the number of scaffolds obtained from each assembly was compared to their average identity with the human genome (hg19 assembly, Supplementary Table S2), a highly significant negative correlation (Pearson correlation coefficient −0.93, p-value 2.379e-06) was found. This observation is consistent with the possibility that an increased number of reads escaping assignment early in the workflow, later resulting in more numerous metagenomics scaffolds, is a reflection of higher levels of somatic mutations and/or genomic instability in tumor samples. Indeed, levels of genomic divergence as high as those recovered in some of our samples are not normally observed in healthy human individuals.

Different samples showed notable disparities in the numbers of metagenomics scaffolds obtained after assembly (Table 2). Interestingly, we notice that the number of scaffolds obtained from RNA samples are consistently higher than from DNA samples. This is more evident when matched RNA and DNA samples from the same specimen (T7) are considered. We speculate that this observation might reflect a general dysregulation of splicing in cancer^16^.

Unsurprisingly, a large proportion (83.82%) of the scaffolds assembled through this strategy showed high levels of identity with human genomic sequences. Conversely, only four samples displayed scaffolds of non-human origin (Table 2). A carcinoma of the tongue and oropharynx (N4) harbored *Fusobacterium nucleatum* RNA. RNA from this bacterium had been already identified by MetaShot in this sample and in a lip tumor (N6, Supplementary Table S1). Three samples showed strong evidence of the presence of viruses. Two tumors (T8 and N6) bore torque teno viruses (TTV) and one (T1) a coxsackievirus. In all three cases, identity with the closest sequence in the database was ≤87%, which suggests that the viruses are new strains and explains why they had not been previously identified by MetaShot, which adopts very stringent sequence similarity thresholds. Interestingly, the scaffolds assembled by metaSPAdes provide complete or nearly complete representations of the viral genomes uncovered by our analyses, indicating the thoroughness of the approach we adopted.

Analyses based on split mapping of the reads, and on spurious mapping of paired reads (see Materials and Methods) provided no evidence of viral integration into the host’s genome. Importantly, we notice that all the metagenomics scaffolds obtained by our assemblies were confidently assigned to a taxon. The observation that no metagenomic scaffolds remained unassigned suggests that it is highly unlikely that significant levels of unrecognized viral sequences were present in our samples. Indeed, we remark that the proportion of reads not assigned to a taxon and not included in any metagenomics scaffolds is consistently low (average ~1.6%, Supplementary Table S2, and see Discussion below).

## Discussion

In this work we analyzed metagenomics data from tumors arisen in immunodeficient patients, in an effort to identify novel, potentially tumorigenic microorganisms associated with human neoplasms. The methods adopted aimed at excluding spurious results and false identifications, while attaining high levels of sensitivity.

For this purpose, we included only tumors diagnosed after ≥3 years from the onset of an immunosuppressive condition and with increased incidences in kidney transplant patients. Surgical tumor fragments were harvested and processed under stringent sterile conditions to prevent microbial contaminations. The transcriptomes and/or genomes of these tumors were subjected to high-throughput sequencing. To attain high levels of sensitivity and specificity, we developed a novel bioinformatics workflow, MetaShot, which performed better than other state-of-the-art pipelines in the characterization of metagenomic samples^14^. Using this tool, we obtained nearly complete representations of three new viral strains associated with our samples, which is an additional indication of the high level of accuracy of the *in silico* analyses performed in this work.

On average, about 1.6% of all reads could not be assigned to a taxon. This proportion might appear high, in light of the fact that tumorigenic viruses can be represented in tumor RNA samples by as few as 2 reads per million^6^. However, it should be stressed that our unassigned reads could not be assembled into scaffolds, making it unlikely that meaningful numbers of them belonged to the same non-human organism. Most probably, they represent human repetitive sequences that cannot be assigned due to ambiguity. Indeed, Supplementary Fig. S1 shows that their compositional profiles (GC content) are virtually identical to that of human satellite sequences. Nonetheless, we cannot completely exclude that nucleic acids from unknown microorganisms were present at low levels in our samples.

Little evidence was found to support the hypothesis that the tumors examined in this work are associated with infectious agents. *Fusobacterium nucleatum* RNA was found in two carcinomas (of the tongue, N4, and of the lip, N6, Supplementary Table S1). This species has been recently linked to colorectal carcinoma^17,18^, where it is suggested to modulate the tumor-immune microenvironment^19^ and alter signaling pathways in the neoplastic cells^3^. Yet, this bacterium is a very common commensal species found preferentially in the oral cavity and therefore its detection bears no special significance. Unsurprisingly, a large number of diverse bacterial taxa were detected in all our specimens. We cannot exclude that some of these species might contribute to, favor, or accelerate tumorigenesis. However, metagenomics approaches cannot identify bacterial species relevant to tumorigenesis without the support of microbiological and epidemiological studies, as the multifaceted tumor/bacteria relationship involves complex molecular interactions, mutagenesis, the microenvironment, and the immune system^3,19^, far from the comparatively simple cause-and-effect paradigm of viral tumorigenesis.

TTV was detected in a lip and a kidney tumor. This virus is the most abundant component of the human virome and is not strongly associated with any pathological condition^20^. A new strain of coxsackievirus was identified in a squamous cell carcinoma of the skin (T1). Although coxsackie viruses cause severe human and animal diseases, they have not been associated with cancer and in fact are sometimes deployed as oncolytic agents. A final caveat is that RNA-seq would not detect integrated but non-transcribed viruses that might promote tumorigenesis, e.g., by deregulating cellular oncogenes. However, no such case was found in the four instances in which tumor DNA was sequenced.

Even if the number of tumors examined in this study may appear small, it should be evaluated in light of the discovery power it affords. Table 3 shows the likelihood of detecting a virus in at least one of the tumors as a function of the number of specimens and the hypothetical virus prevalence. For example, probability calculations show that, in skin tumors (the largest class of cancers investigated, n = 5), detection probability was 96.9% with a hypothetical virus prevalence of 50%. With the same prevalence, there was a 93.8% probability of detection in kidney tumors (n = 4). If one considers all 13 tumors together, as allowed by the fact that they all display increased incidences in immunodeficient persons, detection probability is 94.5% even with a 20% overall virus prevalence. It should be noted that, in the best known cases of direct viral carcinogenesis, viral prevalence in tumors is usually high: HPV, 83–89% in cervical^21^ or 26% in head and neck^22^ cancers; EBV, 15–30% in Burkitt’s lymphomas in the USA, >90% in Africa, >95% in nasopharyngeal carcinomas, and 41–94% in Hodgkin’s lymphomas^23^; MCPyV, 58–100% in Merkel cell carcinomas^24^; HHV8, 100% in Kaposi’s sarcomas^25^.

**Table 3:** Probability to detect the presence of viruses.

% prevalence	% detection probability^1^		
	n = 4	n = 5	n = 13
10	34.39	40.95	74.58
20	59.04	67.23	94.50
30	75.99	83.19	99.03
50	93.75	96.88	99.99
80	99.84	99.97	>99.99

^1^Probability to observe a virus that has the indicated hypothetical prevalence in the tumor group considered, in at least one of n samples. Probability is calculated according to the following formula: D = [1 − (1 − P)^n^] × 100, where D is detection probability and P is prevalence, with 0 ≤ P ≤ 1.

Altogether, our negative findings, along with similar results in the literature (e.g., refs. ^6,8,26^), suggest that unknown tumorigenic viruses are rarer than plausibly hypothesized. Indirectly, they support the possibility that the increased incidence of neoplasia in immunocompromised subjects is, at least in some cases, the result of impaired tumor immune surveillance.

## Materials and Methods

### Patients

Tumor samples were provided by three Italian transplantation units and one cancer center. Only neoplasms from patients in a chronic immunosuppressed condition for at least three years were included in the present study. The 13 tumor samples analyzed in this study came from 12 patients. The samples labeled T12 and T13 (skin carcinomas) were from the same patient. All tumors except the cutaneous ones were the first neoplasms diagnosed after the onset of the immunosuppressive conditions. The project as a whole has been approved by the Ethics Committee of the Istituto Superiore di Sanità. In addition, the ethics committees of all participating clinical centers reviewed and approved the project and approved the information sheets and informed consent forms. All patients provided informed consents to participation in the study.

### Samples and sample preparation

Tumors were surgically removed for therapeutic purposes. Fragments of the neoplasms were obtained in a sterile fashion. Sterility was maintained throughout sample shipment and handling, to prevent contamination by extraneous microorganisms. DNA and RNA were extracted simultaneously from all samples, using the AllPrep DNA/RNA/miRNA Universal kit (Qiagen, Venlo, The Netherlands), following the manufacturer’s instructions; this kit quantitatively retrieves all types of RNA, including small (e.g., miRNAs) and non-polyadenylated RNAs. Nucleic acid quality and quantity was evaluated spectrophotometrically and by agarose gel electrophoresis (DNA) or on a BioAnalyzer (RNA) (RNA 6000 Nano Kit, Agilent, Santa Clara, CA). All the experiments were performed in accordance with the relevant guidelines and regulations.

### Sequencing

RNA samples of sufficient quality (RNA Integrity Number, RIN ≥ 7.0) and quantity were subjected to direct sequencing. Alternatively, when RNA was not deemed satisfactory, genomic DNA was sequenced. Both RNA and DNA from a single sample (T7) were sequenced.

Strand-oriented RNA-Sequencing: for each RNA sample, a directional library was prepared using the TruSeq Stranded Total RNA Sample Prep Kit (Illumina, San Diego, CA, USA), according to the manufacturer’s instructions. Ribosomal RNA depletion was performed using Illumina Ribo-Zero Epicentre kits. The cDNA libraries thus obtained, were checked for quality and quantity and finally sequenced on the Illumina NextSeq500 platform for the target production of 150 M 100-bp PE reads.

Whole genome sequencing: DNA was subjected to library preparation using the TruSeq DNA PCR-free Sample Prep kit (Illumina, San Diego, CA, USA), including inserts from 200 to 500 bp, approximately. The library was sequenced on an Illumina NextSeq500 platform on a 2 × 100 bp PE sequencing run.

In both RNA and DNA sequencing, samples were processed as indexed pools, using NextSeq 500 High output kits v2 (300 cycles).

### Taxonomic profiling

Genomic and transcriptomic NGS data were analyzed by means of the MetaShot^14^ workflow for the characterization of the composition of the microbiome and virome. Low quality reads (Phred score <25) were trimmed using FaQCs^27^; MetaShot we assume that Quality score lower than 25 are associated to low quality region of sequences and consequently we trim them. phi X bacteriophage^28^ contaminant sequences were detected and removed using Bowtie 2^29^. Quality trimmed data were aligned to the human genome release hg19 using STAR^30^ and to a collection of reference genomic assemblies for prokaryotes, fungi, protists, and viruses (obtained from GenBank and RefSeq NCBI nucleotide databases) by means of Bowtie 2. Finally, uniquely mapping sequences were taxonomically annotated using the TANGO (Taxonomic assignment in metagenomics) tool^31,32^ on the NCBI taxonomy.

### Assembly and characterization of unassigned sequences

Reads unassigned by TANGO were assembled using the metaSPAdes metagenomic assembler^15^, with default parameters and the following values for the kmer size parameter: 33, 55, 77, 99, 121. The WindowMasker and RepeatMasker programs were used to annotate human microsatellites and repeats. Scaffolds containing a high proportion of human repeats (greater than 30% of the scaffolds size) and scaffolds shorter than 250 bp in size were excluded from subsequent computations. Sequence similarity searches against the nr refseq database and the complete collection of human transcripts (according to the Gencode 31 annotation of the human genome) were performed with the blastn program (again, with default parameters). In brief, scaffolds for which the alignment with the best blast match covered more than 30% of the scaffold sequence with an average sequence identity of 70% or greater were assigned to their respective best match. Finally, scaffolds not showing significant levels of identity with publicly available sequences, were subjected to manual investigation by performing similarity searches of in silico six-frames translated sequences against the complete viral genome database and the nr database at NCBI, using the tblastx program. We arbitrarily considered only specimens for which more than 1% of the total genome size or more than 10 kb of genomic sequence were covered by metagenomics scaffolds. Microsatellites sequences for the hg19 reference human genome assembly were obtained from the RepeatMasker track of the UCSC genome browser (http://genome.ucsc.edu/), using the table browser. Compositional profiles in Supplementary Fig. S1 were computed by means of a custom Perl script.

### Identification of possible sites of viral integration in the human genome

For all the viral specimens that were identified in our metagenomics assembly, approaches based on split mapping of reads and on incoherent mapping of read pairs were applied, to identify putative viral integration sites in the human genome. All unassigned reads were aligned to the collection of viral metagenomics scaffolds identified in this study and to the hg19 reference assembly of the human genome by means of the Bowtie 2^29^ program, using the “very-sensitive-local” presets. A custom script was subsequently applied in order to identify single reads showing partial similarity (identity ≥ 95% and alignment longer than 40% of the read size) to both the human genome and a viral scaffold or, alternatively, pairs of reads for which one mate could be confidently mapped (completely aligned with an identity level ≥ 95%) to a metagenomics scaffold of viral origin, and the other to the hg19 assembly of the human genome.

### Accession codes

Raw sequencing data are available online at https://www.ncbi.nlm.nih.gov/bioproject/PRJNA544407.

## Supplementary information

Supplementary Figure S1

Supplementary Table S1

Supplementary Table S2
