# Supplementary material for: No metagenomic evidence of tumorigenic viruses in cancers from a selected cohort of immunosuppressed subjects
Source: Sci Rep. 2019 Dec 24;9:19815. doi: 10.1038/s41598-019-56240-1 (PMC6930283; doi:10.1038/s41598-019-56240-1)
Supplement: Supplementary file 1 — Supplementary Figure S1 [file 41598_2019_56240_MOESM1_ESM.pdf]

No metagenomic evidence of tumorigenic viruses in cancers from a selected cohort of immunosuppressed subjects

Nunzia Passaro, Andrea Casagrande, Matteo Chiara, Bruno Fosso, Caterina Manzari, Anna Maria D'Erchia, Samuele Iesari, Francesco Pisani, Antonio Famulari, Patrizia Tulissi, Stefania Mastrosimone, Maria Cristina Maresca, Giuseppe Mercante, Giuseppe Spriano, Giacomo Corrado, Enrico Vizza, Anna Rosa Garbuglia, Maria Rosaria Capobianchi, Carla Mottini, Alessandra Cenci, Marco Tartaglia, Alessandro Nanni Costa, Graziano Pesole, and Marco Crescenzi

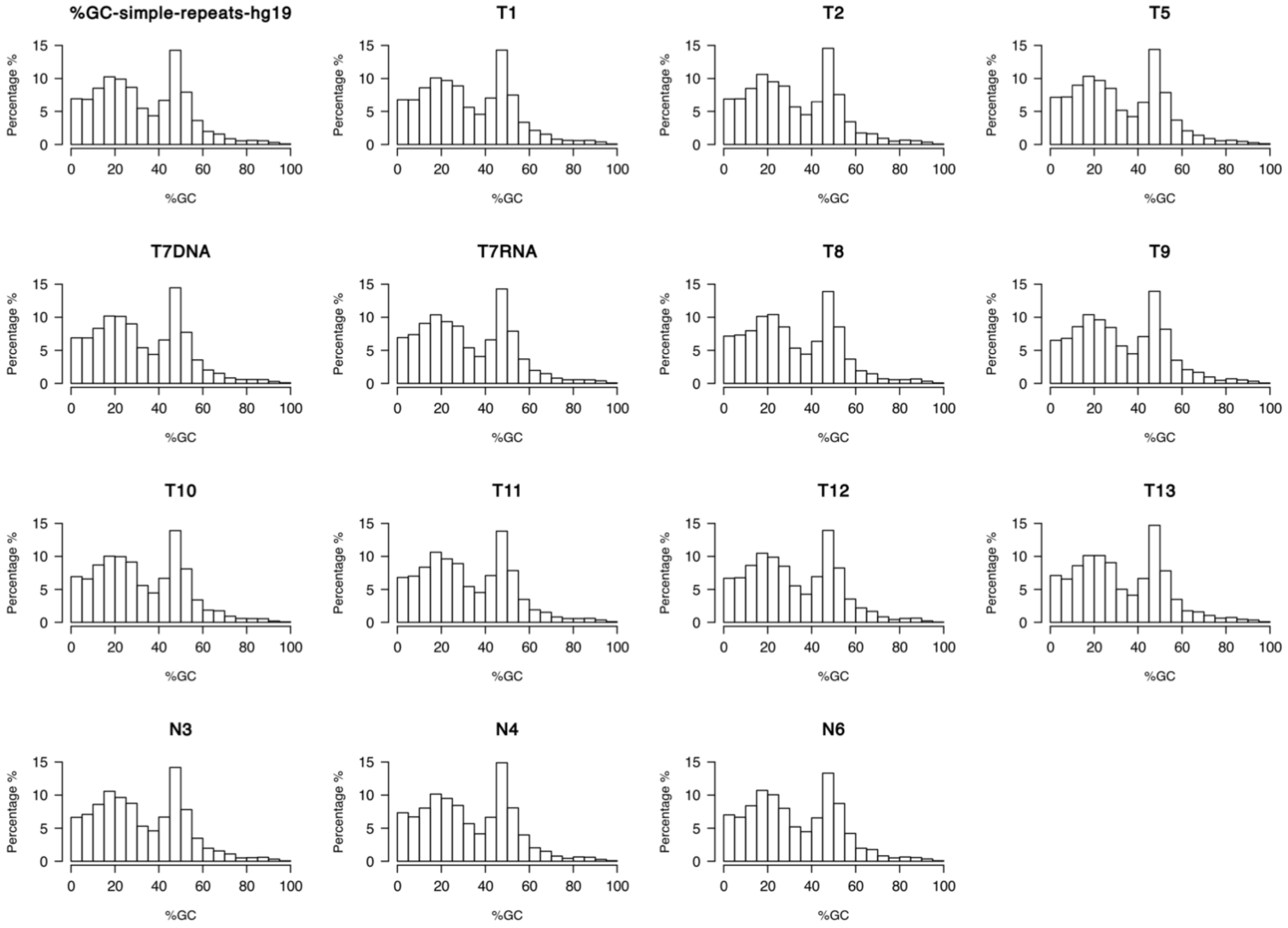

Supplementary Figure S1. Histograms of compositional profiles of simple repeats (microsatellites) of the human genome hg19 reference assembly (top, leftmost panel) and of the unassigned reads from the 14 samples analyzed in this study. Bins of GC composition (20 bins of equal width from 0 to 100%) are represented on the X axis. The frequency of each bin is represented on the Y axis.
